# “The Group Where I Opened My Box”: The Impact of Psychodrama on University Students’ Anxiety, Social-Emotional Competence, and Coping – A Mixed-Method Quasi-Experimental Study

**DOI:** 10.1192/j.eurpsy.2026.10920

**Published:** 2026-07-31

**Authors:** I. Ayvat, E. Ağtaş Ertan, B. Kirlangiç Şimşek

**Affiliations:** 1 Psychiatric Nursing Department, Hacettepe University; 2 Psychiatric Clinic, Bilkent City Hospital; 3 Dr. Abdülkadir Özbek Psychodrama Association, Ankara, Türkiye

## Abstract

**Introduction:**

University students, as Gen Z, are particularly vulnerable to elevated stress, anxiety, and emotional dysregulation during the emerging adulthood period. This phenomenon may have negative effects on academic performance, social interaction, and overall well-being. However, preventive and experiential mental health interventions remain underdeveloped in higher education settings.

**Objectives:**

This study aimed to evaluate the effects of a psychodrama group intervention on anxiety, coping strategies, and socio-emotional competencies among university students.

**Methods:**

This mixed-method quasi-experimental study utilized pre-test, post-test, and follow-up measurements (quantitative) and sociometric techniques in the second and tenth sessions of both groups (qualitative). Participants in the intervention group (n=9) attended ten weekly psychodrama sessions between October 2024 and January 2025, while the control group (n=11) received no intervention. The control group later received the intervention in March–May 2025. Quantitative data were analyzed using Mann-Whitney U (between-group) and Friedman tests with post-hoc Wilcoxon and Bonferroni correction (p<.0167). Thematic analysis (tree game) and content analysis (sociometric spectrograms) were employed to analyze the data.

**Results:**

Quantitative data demonstrated that, compared to the control group, the intervention group demonstrated significant improvements in emotional expressivity, decline in anxiety (BAI), and enhanced coping strategies as Adjustment, and Self-Punishment subscales, with sustained changes in Self-Help at follow-up. Qualitative analysis revealed eight themes, “Growth and Separation from Roots”, “Ability to Build New Bonds”, “Inner Evolution” and “Self-Sufficiency” from tree games”; “Emotion Regulation”, “Socio-Emotional Contact”, “Self-Awareness” and “Flexibility” from sociometric spectrograms.

**Conclusions:**

Integrating quantitative and qualitative findings demonstrates that psychodrama positively impacts students’ emotional, cognitive, and social functioning. The safe and structured group environment enabled authentic emotional expression, discovery of personal resources, and exploration of adaptive coping strategies. These findings support the use of experiential group-based programs in university mental health services. Future large-scale and longitudinal studies are needed to evaluate sustainability, generalizability, and mechanisms of change.

**Disclosure of Interest:**

None Declared

